# Bi-Pinnate Compound *Serianthes nelsonii* Leaf-Level Plasticity Magnifies Leaflet-Level Plasticity

**DOI:** 10.3390/biology9100333

**Published:** 2020-10-13

**Authors:** Benjamin E. Deloso, Thomas E. Marler

**Affiliations:** Western Pacific Tropical Research Center, College of Natural and Applied Sciences, University of Guam, UOG Station, Mangilao, GU 96923, USA; delosob@triton.uog.edu

**Keywords:** acclimation, conservation biology, functional leaf traits, Guam

## Abstract

**Simple Summary:**

Trees are not able to move in order to avoid stressful conditions. Therefore, trees have developed elaborate methods for modifying their organs to benefit the tree under prevailing conditions. For low light and high light conditions, most studies have looked at how the leaf blade is modified. We have shown that the entire leaf should be observed for modifications rather than just the leaf blade.

**Abstract:**

Numerous leaf traits exhibit developmental plasticity in response to irradiance, an attribute that maximizes performance in the prevailing light. The use of leaflets to represent whole leaf traits of tree species with compound leaves is common in the acclimation literature. These methods ignore the potential for whole leaf plasticity to augment leaflet plasticity. We grew *Serianthes nelsonii* plants in incident light ranging from 6% to 100% of sunlight and quantified numerous leaflet and leaf traits to determine plasticity index (PI: (maximum-minimum)/maximum)) of each. Leaflet acclimation such as changes in length of palisade mesophyll occurred as expected. However, leaf-level morphometric traits such as rachillae insertion angle also exhibited acclimation potential. The leaf-level plastic behavior enabled acclimation approaches that simple-leaved species do not possess. We illuminate the need to look at the entire leaf when quantifying acclimation potential of tree leaves, and indicate that the historical use of leaflets to represent species with compound leaves under-estimated the acclimation potential when compared to species with simple leaves.

## 1. Introduction

Light is one of the most influential abiotic resources that plants use to make decisions about growth and development. The allocation, anatomical, biochemical, geometrical, and morphological alterations that occur in plants in response to light signals allow the plants to improve performance in varied forest habitats [1,2,3]. The organ that has been most studied with regards to light is the leaf. Plants benefit by changes in organ traits that enhance photon capture when grown in limited light, and benefit by changes in organ traits that maximize carbon fixation when grown in high light. As a result, plants that are able to acclimate to limited light will produce thin leaves with increased area, using fewer resources to construct greater leaf surface for capturing photons. Available resources are invested into the machinery of the light-harvesting complex [4,5]. The thin laminae in shade leaves also minimize self-shading of the chloroplasts that reside in abaxial cells of the laminae [6]. Moreover, the conservative use of resources for construction of leaf functional components allows those resources to become available for growth in other organs [7]. In contrast, plants that are able to acclimate to high light will produce thick leaves with decreased area, enabling greater photosynthetic capacity per unit leaf area [2]. Smaller leaves exhibit reduced boundary layer thickness, which enables greater convective cooling [8,9,10]. As larger vascular conduits exist in sun, leaves, this also enables greater cooling capacity through increased transpiration capacity [11]. Available resources are invested into the machinery that drives the dark reactions of photosynthesis to maximize use of the non-limiting light levels, but because there is a maximum to which photosynthesis can be increased, the leaf responses also add tools to protect against damage caused by the excessive light [12,13].

Plant leaf forms, shapes, and designs vary greatly among species. The influence of incident light on compound leaf plasticity has been examined by several publications. However, the majority of these studies did not delve into whole leaf traits and instead used a leaflet as a leaf analog to compare with species that produced simple leaves [14,15,16]. The few instances where plasticity of compound leaf morphology traits has been reported were studies of pinnately compound leaves [17,18,19]. To our knowledge, no previous study has provided a detailed look at whole leaf morphometric trait plasticity to incident light for bi-pinnately compound leaves.

The magnitude and nature of acclimation to incident light varies greatly among plant species. The variation in acclimation has been used to lump plant species into binary classifications of sun or shade species [1,20]. Alternatively, pioneer species have been attributed with greater potential for acclimation to changes in incident light because the open habitats exhibit greater variation in abiotic resources than the understory of closed canopy forests [21,22,23]. However, the most plausible viewpoint contends that there is a continuum between sun and shade acclimation potential and the species that produce leaves naturally found in both exposed and shaded habitats may exhibit the greatest acclimation capacity [24]. In particular, late successional species that spend their early years in the forest understory then grow tall and displace neighbors to claim parts of the emergent canopy in late years of ontogeny exhibit acclimation potential that provides a competitive advantage for the species regardless of the stage of ontogeny [25,26,27].

*Serianthes nelsonii* Merr. is a late successional tree species that produces relatively large bi-pinnate compound leaves with numerous small leaflets. The species is characteristic of several tree species from the Mariana Islands in that the declining populations are comprised of large adults with few saplings and juveniles. Recent conservation research has revealed that recruitment is substantial, and the regeneration failures are due to rapid seedling mortality [28,29]. No contemporary seed vector is known, so 100% of observed seedlings emerge under the parent trees where incident light is severely limited. Limited light may be one of the stressors that cause the recruitment failures [30].

The behaviors of tropical forest tree leaves to developmental light characteristics have been heavily studied [9]. However, not all species express plasticity in leaf traits to growth irradiance and context dependency may alter plant responses [31]. Therefore, when knowledge of acclimation potential of a critically endangered species such as *S. nelsonii* [32] is needed to improve conservation decisions, empirical studies are required to generate the species-specific knowledge. The primary aim of this work was to determine which morphological and anatomical leaf traits exhibited the greatest level of plasticity when *S. nelsonii* plants were subjected to a wide range of incident light. This information is of utmost importance for improving conservation decisions of this critically endangered tree species. The secondary aim of this work was to compare leaf-level trait plasticity to leaflet-level trait plasticity to inform future acclimation research methods involving species with compound leaves.

## 2. Materials and Methods

*Serianthes nelsonii* has a limited endemic range that includes the islands of Guam and Rota [32]. The critically endangered legume tree is a member of the Mimosoideae subfamily. The tree is known locally as Håyun lågu and the threats are numerous [30,32]. Known areas of occupation are characterized by alkaline karst soils. Frequent tropical cyclones characterize the endemic range [32]. Quantification of photosynthetic photon flux density (PPFD) beneath a mature *S. nelsonii* tree on Guam was accomplished on several dates in 2014 and 2015 to more fully understand the habitat conditions of in situ seedlings. A 1-cm quantum sensor (SKP200, Skye Instruments, Wales, UK) was used to determine PPFD of numerous shade-flecks and concurrent PPFD of the solar beam on days with varied conditions of sky cover. On the same days, a 75-cm line quantum sensor (EMS-7, PP Systems, Amesbury, MA, USA) was used to quantify integrated PPFD among numerous locations beneath the tree’s canopy and these data were compared with concurrent measurements of PPFD of the solar beam. In situ *S. nelsonii* seedlings emerging beneath a mature tree were subjected to shade that ranged from 1% to 7% of full sunlight when PPFD was quantified with the 1-cm quantum sensor. A typical shade-fleck exhibited PPFD of 60–70 μmol·m^−2^·s^−1^ on clear days. The integrated PPFD as captured by the line quantum sensor indicated a mean of about 6% to 7% of sunlight was typical for these in situ forest floor conditions. We used these field data to define 6% of sunlight transmission as the minimum light for our leaf acclimation study methods.

The leaves for this study were obtained from nursery plants grown at the University of Guam. Nursery protocols conformed to previously described methods [33,34,35]. Seeds were germinated in 10-cm diameter containers in November 2014 under 50% sunlight transmission. The seedlings were moved to 15-cm diameter containers in March 2015 and placed in 6%, 38%, 73%, or 100% of sunlight transmission. Incident light exclusion was provided by commercial nylon shade fabric. Leaf traits were measured in August 2015. The initial response variable was obtained from photographs of the median rachilla of one leaf from each of six plants per light treatment. The youngest fully expanded leaf was employed. This non-destructive procedure used ImageJ open access software [36] to calculate the area of every leaflet from the base to the apex of the rachilla. The outcomes indicated individual leaflet area was heterogeneous along the rachilla axis but exhibited a homogeneous pattern for each of the light treatments. The individual leaflet area (MLA) was calculated as the mean of all leaflets for each replication. All abbreviations and units are shown in Table 1.

Earlier observations and experimental treatments indicated *Serianthes* plants produced with traditional container nursery protocols develop inadequate root growth in relation to shoot growth, and periodic stem tip pruning during nursery production increased relative root growth and mitigated rapid post-transplant mortality [35]. The excised leaves for this study were derived as a byproduct from pruned stem portions that resulted from stem tip pruning of *S. nelsonii* stock in August 2015. The youngest fully expanded leaf from each replication was selected. The plants were 90–110 cm in height with basal stem diameter of 2.1–2.3 cm at the time.

### 2.1. Leaf Measurements

For each light treatment, leaflets were collected from the median position of the median rachilla from 10 replications to use for later microscopy methods. The leaflets were stored in 70% ethyl alcohol until microtome methods were conducted (described in Section 2.3). The youngest fully expanded leaf was obtained from six plants per light treatment. Leaves were immediately photographed with a scale to mm accuracy for later image analysis to determine uni- and two-dimensional leaf and leaflet traits (described in Section 2.2). Determination of leaflet mass per area (LMA) utilized every leaflet on one side of the median rachilla of each leaf, to ensure leaflets of the full range in leaflet area were included. A photograph of the intact rachilla was taken, then the leaflets were removed and combined into one sample. ImageJ software was used to measure the area of every leaflet from the photographs, and the total area was calculated by adding the area of each leaflet. The combined leaflet sample was dried at 75 °C for 24 h then weighed. The LMA (g·m^−2^) was calculated by dividing dry weight by area.

The remainder of the leaflets on each leaf were removed, and the leaflets that were used to determine LMA were added to the whole leaf sample of leaflets. Dry weight of all leaflets (LFW) was determined by weighing after 24 h at 75 °C. Dry weight of support tissue was determined by combining petiole, rachis, and rachilla tissue into one sample and weighing after 24 h at 75 °C (STW).

### 2.2. Photographic and Derived Analyses

**Figure 1:**
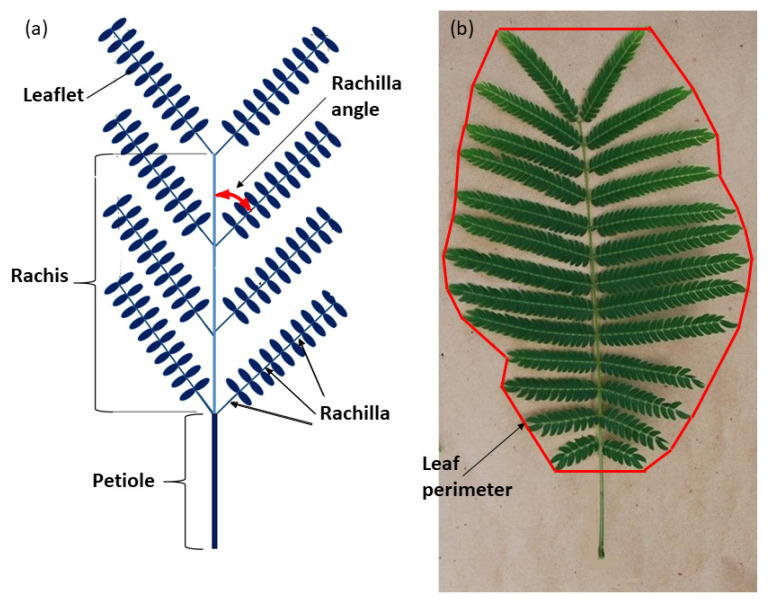
Representative *Serianthes nelsonii* bi-pinnately compound leaf. (**a**) Schematic with key structures labeled. (**b**) Actual leaf showing method of defining leaf perimeter.

The stored whole leaf photographs were analyzed in August 2020. The length of the petiole (PL), rachis (RL), and widest point of the whole leaf (LD) were measured. The number of rachilla per leaf (RPL) was counted and the insertion angles of the rachillae (RA) were measured (Figure 1a). The mean of all rachillae was used as one replication. The total number of leaflets per leaf (LFL) were counted. Maximum rachilla length (MRL) and leaflets per rachilla (LR) were counted from one of the median rachilla from each leaf. Leaflet length (LL) and leaflet diameter (LFD) were measured for every leaflet on the median rachilla and the mean of all leaflets was used as the replication. Area of the two-dimensional space occupied by the entire leaf (PA, cm^2^) was calculated by circumscribing the perimeter of the entire leaf by connecting the end of each rachilla (Figure 1b). ImageJ software was used to calculate the two-dimensional area within the perimeter. Total leaflet area (WLA) for each leaf was calculated by multiplying LFL by MLA. Total leaf weight (TLW) was calculated by adding STW and LFW. The proportion of leaf biomass in the support tissue (SWQ, g·g^−1^) was calculated by the quotient STW/TLW. The relationship of support tissue to leaf area (SAQ, g·m^−2^) was calculated by the quotient STW/WLA. The proportion of PA occupied by actual leaflet area (LFQ, cm^2^·cm^−2^) was calculated by the quotient WLA/PA. The *S. nelsonii* leaf is a bi-pinnately compound organ (Figure 1b). The leaflet of *S. nelsonii* is a homobaric lamina with a midrib. The petiolule is sessile and equipped with a highly active pulvinus. The rachis exhibits paripinnate rachilla arrangement, and each rachilla exhibits paripinnate leaflet arrangement.

### 2.3. Microscopy

The leaflets that were stored in 70% ethyl alcohol for microscopy methods were cleared through a series of 95% ethyl alcohol, isopropyl alcohol, iso-xylene, xylene, then xylene-paraffin. Once cleared, the paraffin additions used standard protocols of 30-min intervals of seven paraffin concentrations until the final paraffin embedding. Once embedded, multiple sections were obtained from the median positions of each leaflet using a Thermo Scientific HM 355S automatic microtome (ThermoFisher Scientific, Waltham, MA, USA). The 8-µm sections were affixed on slides, then stained with Fast Green and counter-stained with Safranin solutions. Finally, the sections were permanently mounted on slides with Canada balsam mounting medium.

Microscopy methods were conducted in August 2020 using a Nikon eclipse 80i digital microscope (Nikon Instruments, Melville, NY, USA). One leaflet section from one leaflet for each of the 10 replications per light treatment was observed, and the thickness of adaxial epidermis (UET), abaxial epidermis (LET), palisade mesophyll (PMT), spongy mesophyll (SMT), and the total leaflet (LFT) were directly measured in three locations per leaflet section. The mean of these three locations was used as one replication, for a total of ten replications. In addition, the thickness of the midvein positioned at the midrib of each leaflet was measured (VT). Palisade to spongy mesophyll relationships were calculated as the quotient palisade thickness/spongy thickness (PSQ, µm·µm^−1^). The relationship of midvein size to leaflet area was calculated as the quotient VT/MLA (VAQ, µm·cm^−2^). Leaflet tissue density (LTD, mg·cm^−3^) was calculated as LFW/(WLA × LFT).

### 2.4. Statistical Analyses

The widely used plasticity index (PI), defined by the difference between the maximum mean and the minimum mean divided by the maximum mean [23], was used to define plasticity of each response variable. Each direct and derived leaf response trait was subjected to analysis of variance (PROC GLM; SAS Institute, Cary, NC, USA). Some of the derived quotients were log-transformed prior to analysis to meet parametric prerequisites. For response variables that exhibited significance, the relationships among the light treatments were plotted in scatter plots to visualize the patterns. The significant response variables exhibited linear or non-linear patterns that deviated from linearity at one extreme of the data range. Therefore, each response variable was subjected to linear and quadratic regression analysis (PROC GLM; SAS Institute) and the model with the greater significance and r^2^ was selected to describe the relationship among the light treatments.

## 3. Results

**Figure 2:**
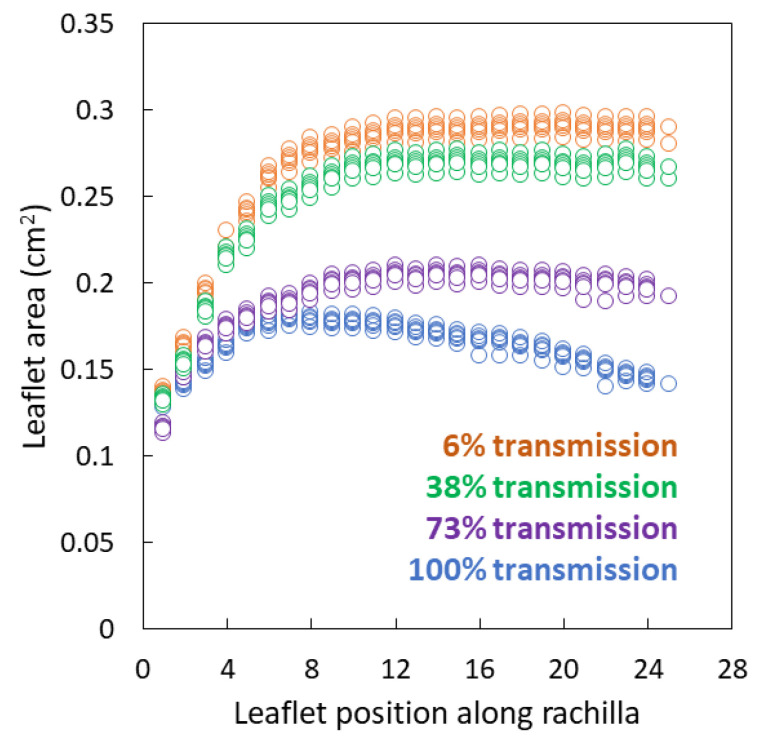
Individual *Serianthes nelsonii* leaflet area from the base to apex of rachillae, as influenced by incident light during leaf growth. Actual data from six replications per light treatment.

Leaflet area was not homogeneous along the rachillae for any of the light treatments (Figure 2). The leaflet area was similar among the light treatments at the base of each rachilla, but began to diverge in area by the third leaflet from the base. For full sun leaves, the leaflet area increased until the 7th or 8th leaflet then gradually declined until the distal leaflet. Maximum leaflet area was 0.17–0.18 cm^2^. For shaded leaves, the leaflet area increased from the base until about 1/3 the length of the rachillae, then remained homogeneous for the distal 2/3 of the rachillae.

### 3.1. Whole Leaf Traits

**Figure 3:**
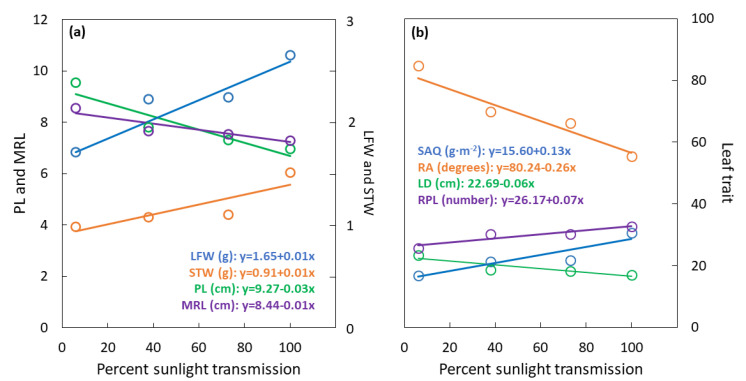
*Serianthes nelsonii* leaf traits as influenced by percent sunlight transmission during leaf development. (**a**) LFW = dry weight of all leaflets, *p* < 0.001, r^2^ = 0.71; STW = dry weight of petiole, rachis, and all rachillae, *p* < 0.001, r^2^ = 0.86; PL = petiole length, *p* < 0.001, r^2^ = 0.87; MRL = maximum rachilla length, *p* < 0.001, r^2^ = 0.84. (**b**) SAQ = STW/area of all leaflets, *p* < 0.001, r^2^ = 0.85; RA = mean angle of rachilla insertion, *p* < 0.001, r^2^ = 0.91; LD = whole leaf diameter, *p* < 0.001, r^2^ = 0.82; RPL = number of rachilla per leaf, *p* < 0.001, r^2^ = 0.85.

The sunlight transmission treatments influenced LFW (F_3,20_ = 28.55; *p* < 0.001) with a substantial linear increase from 1.7 g at 6% to 2.7 g at 100% sunlight transmission (Figure 3a). Similarly, the sunlight transmission treatments influenced STW (F_3,20_ = 15.74; *p* < 0.001) with a modest linear increase from 1 g at 6% to 1.5 g at 100% sunlight transmission. The sunlight transmission treatments influenced PL (F_3,20_ = 22.72; *p* < 0.001) with a linear decrease from 10 cm at 6% to less than 7 cm at 100% sunlight transmission. The sunlight transmission treatments influenced maximum rachilla length (MRL) (F_3,20_ = 9.41; *p* < 0.001) with a linear increase from 8.5 cm at 6% to 7.5 cm at 100% sunlight transmission.

The sunlight transmission treatments influenced LD (F_3,20_ = 50.96; *p* < 0.001) and rachilla insertion angle (RA) (F_3,20_ = 124.32; *p* < 0.001) with linear decreases from 6% to 100% sunlight transmission (Figure 3b). Modest linear increases in SAQ (F_3,20_ = 9.14; *p* < 0.001) and RPL (F_3,20_ = 7.32; *p* = 0.002) occurred from 6% to 100% sunlight transmission.

A significant linear increase in TLW and LFL occurred from 6% to 100% light transmission treatments (Table 2). These traits in leaves from 6% sunlight transmission were 65% to 80% of those in leaves from full sunlight. In contrast, a significant linear decrease in PA occurred from 6% to 100% light transmission, indicating the leaves in shade occupied more two-dimensional space than full-sun leaves while using less biomass to achieve this accomplishment. The length of the rachis and the number of leaflets for each rachilla were similar among the light treatments, indicating the increased PA in shade leaves was not accomplished by rachis length plasticity and the increased LFL in full-sun leaves was not achieved by increasing the leaflets produced on each rachilla. The total area of all leaflets and the LFQ were also similar among the light treatments, indicating the increases in some influential characteristics were counter-balanced by similar decreases in other influential characteristics for these two important leaf traits. The differences in SWQ were significant, but the differences were modest with the lowest value being only 11% lower than the greatest value. Moreover, the relationship with light treatment was not linear, indicating incident light during leaf development may exert a complicated relationship with the amount of total biomass that these leaves allocated to structural tissues.

### 3.2. Leaflet Traits

**Figure 4:**
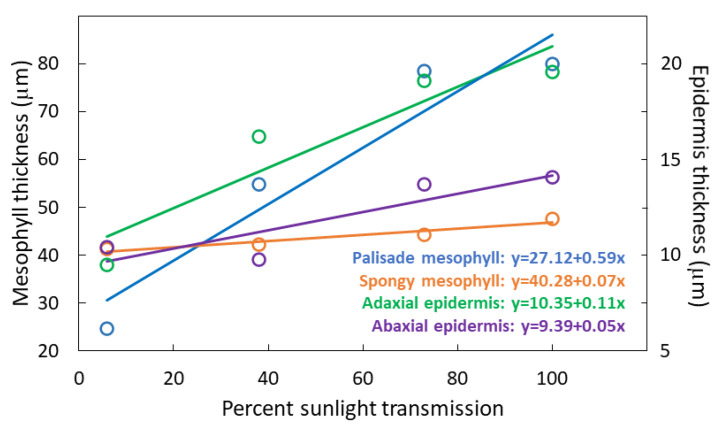
Thickness of four components of *Serianthes nelsonii* leaflets as influenced by percent sunlight during leaf growth. Markers are mean of 10 replications.

The treatments influenced PMT (*F*_3,36_ = 379.04; *p* < 0.001) with a 2.8-fold increase from 6% to 100% sunlight transmission (Figure 4). The treatments influenced SMT (*F*_3,36_ = 4.22; *p* = 0.0120) with a 13% increase from 6% 100% sunlight transmission. The treatments influenced UET (*F*_3,36_ = 47.08; *p* < 0.001) with a doubling of thickness from 6% to 100% sunlight transmission. The treatments influenced abaxial epidermis thickness (LET) (*F*_3,36_ = 9.76; *p* < 0.001) with a 40% increase from 6% to 100% sunlight transmission.

Individual leaflet area, diameter, and length decreased from 6% to 100% light transmission (Table 3). These traits in leaves from 100% sunlight transmission were 70% to 83% of those in leaves from 6% sunlight transmission. In contrast, leaflet lamina thickness of 6% transmission leaves was about half of that for 100% light transmission, and midvein thickness of 6% light transmission leaves was 64% of that from 100% light transmission leaves. The 85% increase in LMA from 6% to 100% sunlight transmission indicated more biomass was required to construct the photosynthetic area of the laminae in full sunlight. The 125% increase in VAQ from 6% to 100% sunlight transmission indicated more vascular tissue was available to service the leaflet’s laminae in full sunlight. The 177% increase in PSQ from 6% to 100% sunlight transmission indicated palisade cells occupied a greater proportion of the total mesophyll tissues in full sunlight leaves. The quadratic regression model was significant for several of the leaflet response variables. However, the linear regression model was equally significant or more significant in every case. The only response variable that was similar among the light treatments was LTD, which averaged about 322 mg·cm^−3^ for these *S. nelsonii* leaflets.

**Figure 5:**
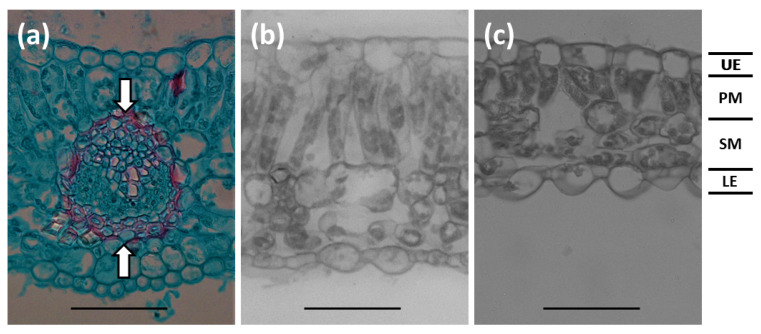
*Serianthes nelsonii* leaflet sections. (**a**) Midvein vascular bundle appearance. Distance between arrows were measured to quantify midvein thickness. (**b**) Section of plants grown in 100% sunlight. (**c**) Section of plants grown in 6% sunlight transmission. UE = adaxial epidermis; PM = palisade mesophyll; SM = spongy mesophyll; LE = abaxial epidermis. Bars = 50 µm.

The appearance of the structure and arrangement of the midvein (Figure 5a) was similar among the light treatments, despite the disparity in vein thickness. The adaxial and abaxial epidermis cells exhibited similar characteristics (Figure 5b,c). Palisade mesophyll cells increased in length from 6% to 100% sunlight transmission. Seldomly, the palisade layer exhibited two cell layers in the 100% sunlight transmission leaflets, but most leaflet sections revealed a single palisade cell layer.

### 3.3. Plasticity

The directly measured leaf traits that were significant exhibited a 5.7-fold range in PI (Table 4). The derived leaf traits that were significant exhibited a 4.5-fold range in PI. The directly measured leaflet traits that were significant exhibited a 5.2-fold range in PI. The derived leaflet traits that were significant exhibited a 1.4-fold range in PI.

## 4. Discussion

This study has revealed a suite of plastic leaf and leaflet traits that *S. nelsonii* plants exploit to acclimate to a range of 6% to 100% incident light during leaf growth conditions. Our results are in general agreement that the substantial leaf plasticity of *S. nelsonii* leaves in response to developmental light verifies considerable acclimation potential as a representative of late successional species that spends early years in the forest understory then grows tall to displace neighbors to claim parts of the emergent canopy in late years of ontogeny [25,26,27]. Our results also address the fact that most of the literature on this subject has focused exclusively on laminae traits. We have revealed that this approach may have accurately characterized simple-leaved species, but it inaccurately characterized compound-leaved species.

### 4.1. Leaflet Versus Whole Leaf

Leaf traits that exhibit greater plasticity may be more critical for leaf functioning in varied light environments [9], and the acclimation attributes enable addition of leaves that perform best in the light environments in which they were constructed [37]. We have revealed a previously unreported repertoire of traits that bi-pinnately compound leaves may exploit while acclimating to growth irradiance. In our study, plasticity of whole leaf traits was extensive and magnified that of leaflet traits. For example, shaded leaves inserted their rachillae close to perpendicular, which spread out the placement of assimilatory surfaces of the leaflets over increased PA. Furthermore, sun leaves produced more leaflets per leaf by producing more rachilla per leaf, not by producing more leaflets per rachilla. Rachis length and total area of leaflets did not differ among the light transmission treatments, indicating the shade leaves employed more distance between the rachilla along the rachis to further spread out the leaflets into the larger PA. Moreover, a plastic PL was used by shade leaves to position leaflets at greater distances from the stem. The WLA was biologically conserved and did not significantly differ among the light treatments, but sun leaves improved thermoregulation by splitting the WLA into more leaflets per leaf, thereby reducing boundary layer thickness of each lamina to enable greater convective cooling [8,9,10]. Simple leaves do not have at their disposal these leaf-level behaviors of compound leaves which can directly influence acclimation success.

These examples of our findings reveal that past publications in which leaflets were used to represent how a tree species (e.g., [14,15,16]) with compound leaves acclimated to varied light conditions underestimated the ability of the leaf to acclimate. In those studies, the range in plasticity for the simple leaf species was accurate, but the range in plasticity for the compound-leaved species was inaccurate. These acclimation skills are remarkable considering that some of them are achieved without any clear costs. For example, changing the angle of a rachillae on a rachis appears to be achievable without any apparent tradeoff in costs. The creation of more distance between leaflets into a larger PA of shade leaves was achieved by increasing the space between fewer rachillae, which also required less biomass for structural leaf components.

Our results have also shown that position of a leaflet within the compound leaf structure may influence the relative area of that leaflet, revealing the mandate to describe which leaflet in compound leaves is selected for acclimation studies. To our knowledge, the literature has ignored this fact in that the position of which leaflet was used to represent trees with compound leaves was rarely included in the published methods. As a result, readers are unable to repeat the methods from these papers because this crucial information was missing.

### 4.2. Benefits of Acclimation

Leaf traits that demonstrate plasticity during acclimation to contrasting growth light conditions may maximize per leaf carbon gain. The ability to alter lamina thickness has been shown to maximize carbon gain per unit leaf mass to a greater degree than altering the allocation of available nitrogen among leaf constituents [5]. Therefore, plastic control of LMA and its inverse specific leaf area are of critical importance for acclimation of trees throughout ontogeny [38]. Our LMA was highly plastic and ranged from 29–54 g·m^−2^, positioning *S. nelsonii* at the low end of the typical range reported for humid tropical forest species (e.g., [39,40]). High light growth conditions increase LMA by increasing the lamina thickness. Indeed, lamina thickness was also a highly plastic trait in our study, ranging from 86–160 µm. Greater leaf thickness in plants grown in high light conditions is partly due to extra layers of palisade cells and/or longer palisade cells [41]. We have demonstrated that the number of palisade cell layers in *S. nelsonii* leaflets is biologically conserved, but the length of the single layer of palisade cells is highly plastic. Leaves may also increase LMA by tightly packing the cell positions to produce minimal air space, which increases density of the lamina tissue [42]. Our results indicate that *S. nelsonii* leaves do not use changes in tissue density to modify LMA, as there were no differences in LTD among the light treatments.

The various components of LMA also influence leaf temperature relations. Stacking the biomass into more layers within limited area passively improves thermoregulation potential because smaller object areas exhibit increases in boundary layer conductance [8,9,10]. Therefore, smaller leaflets in full sun conditions enable greater cooling potential, which would benefit the emergent canopy leaves of a large tree but would not be needed by the leaves in the lower shaded strata. The petiolule of *S. nelsonii* leaflets is equipped with a highly active pulvinus that generates paraheliotropic movements during diurnal periods and nyctinastic movements during nocturnal periods [43]. The protective capacity of this ability to use laminae movement to avoid the solar beam is substantial for full-sun leaves, reducing leaflet temperature and photoinhibition as quantified by chlorophyll fluorescence.

### 4.3. Conservation Biology

The current level of knowledge indicates that developing a greater understanding of how *S. nelsonii* seedlings and saplings persist in the forest understory is mandatory for improving conservation decisions designed to mitigate the lack of recruitment in natural populations and reverse the decades of mortality after out-planting of managed conservation plantings. Indeed, understanding differences in minimum light requirements and relative shade tolerance among co-occurring species is crucial for developing realistic predictions about forest dynamics [44]. The 1994 recovery plan for *S. nelsonii* included establishment of four populations of 500 mature trees on Guam, and the plan estimated 16 years would be needed to reach this goal [45]. To date, there have been no successes toward that goal, and the attempts to establish mature trees through anthropogenic actions have failed. Therefore, expanding our knowledge of how the full range in natural incident light influences *S. nelsonii* leaf and whole plant behavior will help conservationists to develop management protocols for improving conservation goals. Our results indicate that forest canopy cover management needs to become an integral component of conservation planning as more knowledge becomes available about the optimum level of incident light for every ontogenetic stage of this critically endangered tree species.

For managed in situ populations, there is no reason for conservationists to waste seeds by allowing natural seed rain and germination beneath the source tree’s canopy. Years of research have revealed that these seedlings exhibit 100% mortality [28,29]. Using pruning to thin the canopy to determine if limited light is causal of the in situ seedling mortality is not possible if the seedlings are growing beneath the canopy of an endangered tree. Therefore, we recommend the planting of seeds in microsites nearby mature *S. nelsonii* trees in Guam and Rota where the emergent canopy is comprised of non-threatened native tree species and the upper strata can be selectively pruned to increase sunlight transmission. Providing seedlings with a range of 25% to 50% sunlight transmission may provide a simple test to determine if the 6%–7% sunlight transmission that we recorded beneath a mature *S. nelsonii* tree is partly causal for the lack of recruitment. These tests would also remove other Janzen-Connell [46,47] stressors that are associated with the zone immediately beneath the source tree’s canopy.

International recommendations for plant species conservation call for the use of non-destructive experimental approaches within conservation projects to enable co-production of new knowledge [48,49,50]. Conserving at-risk living species such as *S. nelsonii* that reside within lands that are under the custody of the United States military calls for the use of scientifically-based management [51]. Our study is an example of how threatened plant species may be managed in a manner that asks pertinent questions about the plants through non-destructive methods.

### 4.4. Future Directions

This first look at how leaflet versus whole leaf traits compare in their plasticity potential for bi-pinnate compound leaves illuminates several avenues of continued research needs.

First, we looked at leaves from one ontogenetic stage, so the methods should be repeated to determine plasticity in leaf traits among individuals of different ontogenetic stages (e.g., [26,52,53]). This can be accommodated by suspending container-grown seedlings in the highest stratum of the forest canopy, and by using shade cloth cages to cover leaves of mature trees that are positioned at the highest stratum of the canopy.

Second, we used nylon fabric to exclude incident light in accordance with established protocols, thereby modifying PPFD without modifying other light characteristics. The methods should be repeated to determine the role of light quality, light direction, and light duration [54] on leaflet and leaf acclimation behaviors.

## 5. Conclusions

*Serianthes nelsonii* plants were grown in incident light ranging from 6% to 100% of sunlight to determine plasticity of leaflet and leaf traits. Leaflet acclimation was revealed in leaflet traits such as length of palisade mesophyll cells. Additionally, leaf-level morphometric traits also exhibited acclimation potential such as the rachillae insertion angle, leaf component dry weights, and the quotient defined by leaf dry weight/leaf area. Our approach revealed how co-production of new knowledge may be achieved when managing threatened species. The results indicate the entire leaf should be studied when quantifying acclimation potential of tree leaves. The historical use of leaflets to represent species with compound leaves underestimated the acclimation potential when compared to species with simple leaves.

## Figures and Tables

**Table 1:** Abbreviations and units for *Serianthes nelsonii* leaf and leaflet traits.

Abbreviation	Trait	Units
LET	Abaxial epidermis thickness	µm
LFD	Leaflet diameter	mm
LFL	Leaflets per leaf	number
LFQ	WLA/PA	cm^2^·cm^−2^
LFT	Leaflet thickness	µm
LFW	Total leaflet dry weight	g
LL	Leaflet length	mm
LMA	Leaflet mass per area	g·m^−2^
LR	Leaflets per rachilla	Number
LTD	Leaflet tissue density	mg·cm^−3^
MLA	Individual leaflet area	cm^2^
MRL	Maximum rachilla length	cm
PA	Area within leaf perimeter	cm^2^
PL	Petiole length	cm
PMT	Palisade mesophyll thickness	µm
PSQ	PMT/SMT	µm·µm^−1^
RA	Rachilla insertion angle	°
RL	Rachis length	cm
RPL	Rachilla per leaf	Number
SAQ	STW/WLA	g·m^−2^
SMT	Spongy mesophyll thickness	µm
STW	Support tissue dry weight	g
SWQ	STW/TLW	g·g^−1^
TLW	Total leaf weight	g
UET	Adaxial epidermis thickness	µm
VAQ	VT/MLA	µm·cm^−2^
VT	Midvein thickness	µm
WLA	Total area of leaflets	cm^2^

**Table 2:** *Serianthes nelsonii* leaf traits as influenced by percent sunlight transmission during leaf growth. Mean ± standard error.

Leaf Trait ^1^	6% Transmission	38% Transmission	73% Transmission	100% Transmission	Significance ^2^
Total leaf wt (g)	2.70 ± 0.18	3.14 ± 0.17	3.33 ± 0.18	4.16 ± 0.20	<0.001; L ***
Leaflets/leaf	1217 ± 36	1426 ± 35	1444 ± 39	1504 ± 46	0.003; L ***
Rachis length (cm)	45.4 ± 1.7	46.2 ± 1.2	44.1 ± 1.7	45.0 ± 1.3	0.538
LR	47 ± 1	47 ± 1	45 ± 1	45 ± 1	0.610
PA (cm^2^)	768.0 ± 19.6	670.7 ± 15.2	649.3 ± 14.9	602.0 ± 14.6	<0.001; L ***
WLA (cm^2^)	293.5 ± 17.4	270.4 ± 19.0	254.6 ± 16.9	251.8 ± 14.1	0.086
LFQ (cm^2^·cm^−2^)	0.38 ± 0.03	0.40 ± 0.02	0.40 ± 0.03	0.42 ± 0.03	0.660
SWQ (g·g^−1^)	0.37 ± 0.01	0.35 ± 0.01	0.33 ± 0.01	0.36 ± 0.01	0.002; Q **

^1^ LR = leaflets per rachilla; PA = Area within perimeter of leaf shape; WLA = Total area of leaflets; LFQ = WLA/PA; SWQ = Support tissue wt/total leaf wt. ^2^ *p* value from ANOVA; L = significance level of linear regression, Q = significance level of quadratic regression; ** = 0.01, *** = 0.001.

**Table 3:** *Serianthes nelsonii* leaflet traits as influenced by percent sunlight transmission during leaf growth. Mean ± standard error.

Leaflet Trait ^1^	6% Transmission	38% Transmission	73% Transmission	100% Transmission	*p* Value ^2^
Area (cm^2^)	0.24 ± 0.01	0.19 ± 0.01	0.18 ± 0.01	0.17 ± 0.01	<0.001; L ***; Q *
Thickness (µm)	85.94 ± 1.39	122.97 ± 1.48	155.50 ± 1.38	159.59 ± 1.63	<0.001; L ***; Q ***
Diameter (mm)	4.33 ± 0.21	3.83 ± 0.18	3.63 ± 0.14	3.13 ± 0.11	<0.001; L ***
Length (mm)	9.57 ± 0.30	8.23 ± 0.28	7.97 ± 0.18	7.52 ± 0.10	<0.001; L ***; Q *
Vein thickness (µm)	44.53 ± 1.85	63.33 ± 1.68	64.33 ± 1.82	69.58 ± 1.55	<0.001; L ***; Q ***
LMA (g·m^−2^)	29.12 ± 2.54	39.80 ± 3.42	44.65 ± 3.77	53.83 ± 3.33	<0.001; L ***
LTD (mg·cm^−3^)	338.88 ± 29.63	323.69 ± 27.85	287.17 ± 24.58	337.33 ± 20.85	0.411
VAQ (µm·cm^−2^)	187.76 ± 6.06	333.19 ± 5.49	368.65 ± 5.07	421.84 ± 23.07	<0.001; L ***; Q ***
PSQ (µm·µm^−1^)	0.60 ± 0.03	1.31 ± 0.05	1.80 ± 0.09	1.66 ± 0.08	<0.001; L ***; Q ***

^1^ LMA = Leaf mass/area; LTD = Leaflet tissue density; VAQ = Vein thickness/area; PSQ = Palisade mesophyll/spongy mesophyll. ^2^ L = significance level of linear regression, Q = significance level of quadratic regression; * = 0.05, *** = 0.001.

**Table 4:** Plasticity index (PI: maximum mean—minimum mean/maximum mean) of *Serianthes nelsonii* leaf and leaflet traits that were significantly influenced by percent sunlight transmission during leaf growth.

Leaf Traits	PI	Leaflet Traits	PI
Perimeter area	0.22	Individual Area	0.29
Total leaf diameter	0.28	Length	0.21
Petiole length	0.26	Diameter	0.28
Rachilla length	0.15	Lamina thickness	0.46
Rachilla number	0.22	Palisade thickness	0.68
Leaflets/leaf	0.19	Spongy thickness	0.13
Rachilla angle	0.35	Adaxial epidermis thickness	0.52
STW ^1^	0.35	Abaxial epidermis thickness	0.46
LFW	0.35	Midvein thickness	0.36
TLW	0.35	LMA	0.46
SWQ	0.10	VAQ	0.55
SAQ	0.45	PSQ	0.64

^1^ STW = support tissue dry weight; LFW = leaflet dry weight; TLW = total leaf dry weight; SWQ = STW/TLW; SAQ = STW/total leaflet area; LMA = Leaf mass/area; LTD = Leaflet tissue density; VAQ = Vein thickness/area; PSQ = Palisade mesophyll/spongy mesophyll.
